# Positive Youth Development, Mental Stress and Life Satisfaction in Middle School and High School Students in Portugal: Outcomes on Stress, Anxiety and Depression

**DOI:** 10.3390/children11060681

**Published:** 2024-06-03

**Authors:** Marina Carvalho, Cátia Branquinho, Barbara Moraes, Ana Cerqueira, Gina Tomé, Catarina Noronha, Tânia Gaspar, Nuno Rodrigues, Margarida Gaspar de Matos

**Affiliations:** 1 ISAMB/Environmental Health/Medical School, University of Lisbon, 1649-004 Lisboa, Portugal; marina.carvalho@ismat.pt (M.C.); catiasofiabranquinho@gmail.com (C.B.); barbaracsmoraes@hotmail.com (B.M.); cerqueira.apm@gmail.com (A.C.); ginatome@sapo.pt (G.T.); catarinanoronha26@live.com.pt (C.N.); tania.gaspar.barra@gmail.com (T.G.); 2 Centro Hospitalar Universitário do Algarve, 8500-338 Portimão, Portugal; 3 Instituto Superior Manuel Teixeira Gomes, 8500-656 Portimão, Portugal; 4 Católica Research Centre for Psychological, Family and Social Well-Being, Faculdade de Ciências Humanas, University Católica Portuguesa, 1649-023 Lisbon, Portugal; 5 Life Sciences Department, University Centre of Lisboa, University Lusófona de Humanidades e Tecnologias, 1749-024 Lisboa, Portugal; 6 Direção Geral de Estatísticas da Educação e Ciência, 1200-774 Lisboa, Portugal; nuno.rodrigues@dgeec.medu.pt; 7 Applied Psychology Research Centre Capabilities and Inclusion, Instituto Superior de Psicologia Aplicada, 1140-041 Lisboa, Portugal

**Keywords:** positive youth development, mental distress, life satisfaction, stress, anxiety, depression, middle school, high school, students

## Abstract

The relation between positive youth development and stress, anxiety and depression was studied considering the mediator role of psychological symptoms and life satisfaction. A total of 3109 students included in the “Psychological Health and Well-being” study of the School Observatory participated, including 1618 females and 1491 males aged between 11 and 18 years old (*M* = 14.45; *SD* = 1.88), belonging to different school groups in different regions of the country. Adolescents with higher stress, depression and anxiety levels reported lower levels of competence, confidence and connection, as well as more psychological symptoms and lower life satisfaction. The results also showed that adolescents with higher levels of competence, confidence and connection reported less psychological symptoms and more life satisfaction. Two separate mediation analyses were performed to analyse the role of mental distress and life satisfaction on the relationship between positive youth development indicators and stress, anxiety and depression. These analyses confirmed the predicted relationships and partial mediations between mental distress and life satisfaction. These results should be taken into account in public policies concerning young people’s health and education that should involve both targeted and indicated prevention strategies, including school and community-based interventions, in order to be effective.

## 1. Introduction

Adolescent development is marked by various challenges, including biological, cognitive, emotional and social changes. The increasing complexity of coping strategies used to adjust to the COVID-19 pandemic represented one such challenge for adolescents, which adversely affected their well-being and academic and interpersonal lives [1,2,3].

Both national and international research studies have underscored that adolescents and young adults encounter various mental health difficulties. In 2022, the Mental Health and Wellness Observatory of the Directorate-General of Education (details related to the Observatory are available online at https://www.dgeec.medu.pt/p/educacao-pre-escolar-basico-e-secundario/observatorios/observatorio-da-saude-psicologica-e-do-bem-estar (accessed on 17 May 2024)) conducted a study that found that over a third of Portuguese students displayed symptoms of psychological distress, including sadness, irritability, nervousness and a deficit of social–emotional skills. According to the World Health Organization [4], approximately one in every seven adolescents worldwide experiences a mental health disorder. Depression and anxiety are among the most prevalent conditions and are leading causes of disability in young people. On an international level, a study conducted during the pandemic reported that 38% of adolescents had depressive symptoms, 20% had anxiety symptoms, and 21% had experienced suicidal and self-harm ideation [5]. Plus, a comparative meta-analysis revealed an increase in psychological symptoms among children and adolescents following COVID-19 [6]. The results obtained through a study developed by Foster et al. [7] showed that stress levels experienced during the early stages of the pandemic led to long-lasting impacts on mental health and an increased risk of internalizing symptoms.

The literature on this topic also outlines the main factors contributing to this risk, which include being female [8], having pre-existing mental health conditions, single-parent households, lower socioeconomic status, a history of maltreatment, lifestyle, social isolation, low resilience, loneliness, stressful life events and family/school stressors [5,6,8]. These indicators seem more pronounced in girls and older students, who represent, in the literature, the groups with more psychological symptoms, lower life satisfaction, lower quality of life and fewer social–emotional skills [9,10,11]. In addition, it is frequently observed that girls present a higher risk of internalizing symptoms [9,12,13].

On the other hand, the literature on positive youth development [14,15], which refers to the ability to prevent mental health problems and promote psychological well-being, has found that girls report higher levels of connectedness and character, and better caring skills, while boys report higher levels of competence and confidence, as well as better psychological adjustment [15,16,17,18,19]. This double pattern demonstrates that girls report lower self-esteem, a poorer self-concept and lower overall well-being, resulting in a less positive self-perception [17,18]. Conversely, boys tend to exhibit enhanced psychological resilience, enabling them to cope effectively with daily challenges [18]. Masselink and colleagues [19] also found that having low self-esteem during early adolescence could increase the likelihood of experiencing depressive symptoms in the future, influenced by avoidance motivation and social challenges. Plus, higher levels of socio-emotional well-being have been found to lead to better mental health and improved life satisfaction [20]. PYD indicators were found to lead to less mental distress, stress, anxiety and depression, and greater mental well-being [20].

These results show that it is crucial to identify and implement early prevention and intervention strategies that focus on identifying risk factors and clinical indicators. However, to our knowledge, there are no studies on the role of individual factors on the relationship between positive youth development indicators and stress, anxiety and depression in adolescents. Thus, the main goal of the present study was to analyse the relationship between these variables, considering the mediator role of psychological symptoms and life satisfaction individually. We expected the indicators of PYD to present a negative effect on mental distress and stress, anxiety and depression. We also expected the indicators of PYD to present a positive effect on life satisfaction. Finally, we expected that mental distress and life satisfaction would mediate the relationship between PYD indicators and stress, anxiety and depression.

## 2. Materials and Methods

### 2.1. Participants

A total of 3109 students, 1618 girls and 1491 boys aged between 11 and 18 years old (*M* = 14.45; *SD* = 1.88), attending grades 7 to 12 in school groups within all regions of the country, participated in this research. Complementary information about the sample can be obtained from Matos et al. [21].

### 2.2. Measures

The present study is part of the general study “Psychological Health and Well-being|School Observatory” commissioned by the Portuguese Ministry of Education and developed with the main aim of studying the main factors related to psychological health and well-being in three age groups of students: preschool and elementary school (reports by teachers/educators); middle school (self-reports); and high school students (self-reports). Additionally, data were collected from teachers (self-report).

For the present study, only data from middle school and high school students were used. The research protocol included socio-demographic questions, related to gender and age, and individual indicators, related to psychological health and well-being, which were assessed by the Portuguese versions of the Positive Youth Development, the Depression, Anxiety and Stress Scale, HBSC-5-short and the Cantril Life Satisfaction Scale.

Positive youth development (PYD) was assessed through three of the dimensions of the PYD scale [22,23]: competence, confidence and connection, composed of 6, 6 and 8 items, respectively. Answers to each of the items are given in a 5-point Likert scale (0 = strongly disagree and 4 = strongly agree, and 0 = never true and 4 = always true). Higher results indicate higher levels of competence, confidence and connection. Adequate internal consistency values, Cronbach’s α, were reported in the original and in the adapted version, varying from 0.80 to 0.92 and 0.80 to 0.87, respectively. In this sample, Cronbach’s α varied from 0.82 to 0.90 (see Table 1).

Depression, anxiety and stress were assessed using the Depression, Anxiety and Stress Scale (DASS-21) [24,25,26], a self-report composed of 21 items combined in 3 dimensions. Participants responded to the items using a 4-point Likert scale (0 = does not apply to me at all and 3 = applies to me most of the time). Adequate internal consistency values were obtained in the original and in the adapted version and varied from 0.84 to 0.89 and from 0.74 to 0.78, respectively. In this sample, Cronbach’s α varied from 0.86 to 0.90 (see Table 1).

The assessment of symptoms of psychological distress [27,28] involved the use of a 5-point Likert scale (1 = rarely or never and 5 = almost every day) consisting of five items. Higher results indicate more symptoms. In this sample, Cronbach’s α was equal to 0.82, evidencing an adequate internal consistency value (see Table 1).

Life satisfaction was assessed using the Cantril et al. scale [29], which involved participants rating their perception of their own life on a scale ranging from 0 to 10, from the worst possible life to the greatest possible life for the individual.

### 2.3. Procedure

The research “Psychological Health and Well-being|School Observatory” was developed under the aegis of the Portuguese Ministry of Education, and was developed through a partnership between the Directorate-General for Education and Science Statistics, the Directorate-General of Education, the National Program for the Promotion of School Success, the Portuguese Psychologists Association, the Calouste Gulbenkian Foundation and the Aventura Social Team/ISAMB, the University of Lisbon (scientific coordinator). Starting in December 2021, the general research had the main goal of assessing and mitigating the negative impact of the COVID-19 pandemic on child and adolescent psychological health, and was developed using stratified and random selection sampling methods within public school groupings in mainland Portugal, as defined by NUTS III (Nomenclature of Territorial Units for Statistical Purposes). All the school groups were contacted by email in January 2022; the ones unavailable to participate were replaced following a new selection, and the procedure was repeated until an agreement was obtained from another school grouping of the corresponding NUTS. Once the schools had accepted, the same stratified and random selection methodology was applied to the class selection process.

Data collection was carried out between February and March using the computers in the schools’ information technology laboratories. Data collection was facilitated by two professional groups from the participating school groupings, including both teachers and psychologists. Responses to the study required prior authorisation from parents/caregivers, who had to accept the informed consent information included in the online data collection instruments. The complete protocol took, on average, 20–30 min. More information about the study procedures is available online at www.dgeec.mec.pt/np4/1357.html (accessed on 21 April 2024).

### 2.4. Statistical Procedures

Data were analysed using SPSS 25.0 (SPSS, Chicago IL, USA) and JASP 0.18.1 (JASP Team, 2023). Correlation analyses were performed through Pearson’s correlation coefficients, with the main goal of verifying the relationships between the studied variables. Subsequently, two mediation analyses were carried out, including one directed to test each of the mediator models (mental distress and life satisfaction), in order to analyse their individual role in the relationship between the positive youth development indicators and DASS dimensions. The basic assumptions for carrying out correlation and mediation analyses were considered. The Maximum Likelihood Estimator method was used in both analyses because all variables presented a normal distribution (See Table 1). Mediation analyses were carried out following the mediation analysis procedures of Baron and Kenny [30], and correlation size effects were interpreted according to Cohen [31]. Specifically, sample size followed the criteria from Cohen [31] and Maroco [32] concerning the sample required for performing mediation analysis. A confidence level of 95% was considered for each of the analyses.

## 3. Results

### 3.1. Relationships between Stress, Anxiety and Depression with Life Satisfaction, Mental Distress and PYD Indicators

Table 2 presents the Pearson correlation coefficients between the studied variables, which present medium to large size effects.

All the DASS dimensions correlated significantly and negatively with the PYD indicators and life satisfaction, with medium (r > 0.40) coefficient values varying between −0.61 (depression and confidence) and −0.41 (stress and competence; anxiety and connection), all significant for *p* ≤ 0.001. Significant medium (r > 0.60) correlations were also observed between the three DASS dimensions and mental distress, varying between −0.49 (anxiety) and −0.60 (depression), all significant for *p* ≤ 0.001. Furthermore, all PYD dimensions correlated significantly with life satisfaction and mental distress, with coefficient values varying from 0.43 (competence and life satisfaction) to 0.55 (confidence and mental distress), all significant for *p* ≤ 0.001.

The obtained results showed that adolescents with higher stress levels and more depressive and anxious symptoms reported lower levels of competence, confidence and connection, higher mental distress signs and lower life satisfaction. The results also showed that adolescents with higher competence, confidence and connection reported higher life satisfaction and lower mental distress (see Table 2).

### 3.2. The Mediator Role of Psychological Symptoms and Life Satisfaction on the Relationship between PYD and DASS Indicators

Two separate mediation analyses were then performed to understand the role of mental distress and life satisfaction on the relation between PYD indicators and DASS dimensions.

**Figure 1:**
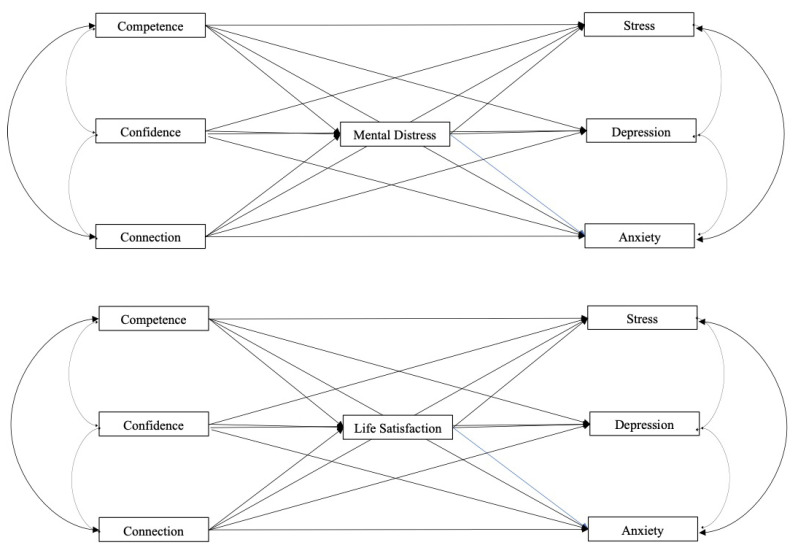
Conceptual mediator model of mental distress and life satisfaction.

The first model analysed the relationship between the three PYD indicators (IVs) and the three DASS dimensions (DVs) considering the mediator role of mental distress (MV). The second model analysed the relationship between the three PYD (IVs) and the three DASS dimensions (DVs) considering the mediator role of life satisfaction (MV) (see Figure 1).

A total mediation effect was considered if (a) PYD dimensions (IVs) significantly predicted the DASS dimensions (DVs); (b) PYD dimensions (IVs) significantly predicted mental distress or life satisfaction (DV); (c) mental distress or life satisfaction (IV) significantly predicted the DASS dimensions (DVs); and (d) if the total effect was reduced to non-significant when mental distress or life satisfaction (MD) was included in the relationship between the PYD indicators and the DASS dimensions. Furthermore, it was considered that the indirect effect of the mediational analysis should be significant and, according to the Sobel test, superior to zero (z > 1.96). A partial mediation effect was considered if the direct effect of this last step was kept significant.

**Figure 2:**
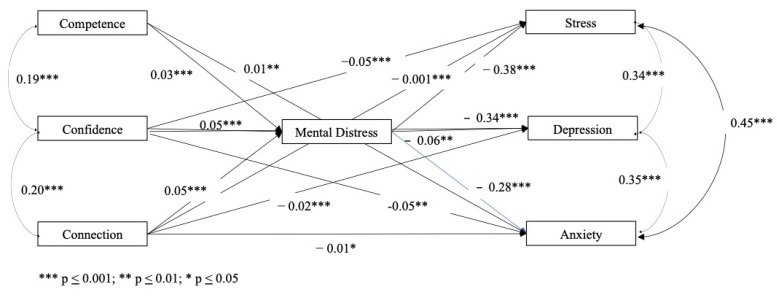
Results of the mediator model for mental distress.

The obtained results for the mental distress mediator model can be observed in Table 3. The significant coefficient paths are presented in Figure 2.

In the mental distress mediation model, when testing the direct and indirect effects of PYD on DASS dimensions, all PYD indicators showed to be significant predictors of mental distress and mental distress was a significant predictor of all DASS dimensions. However, the PYD competence indicator only predicted anxiety whereas PYD confidence and connection were predictors of all DASS dimensions (see Table 3).

As expected, significant intercorrelations within PYD and DASS dimensions were observed in the obtained partial mediation model (see Figure 2), in which mental distress explained 39% of the total variance and the other endogenous variables explained 32% (anxiety), 37% (stress) and 48% (depression).

**Figure 3:**
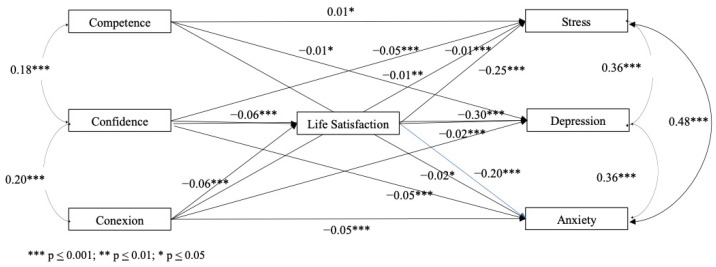
Results of the mediator model for life satisfaction.

The estimates of the effects obtained for the life satisfaction mediator model are presented in Table 4. The significant coefficient paths are also presented in Figure 3.

In the life satisfaction mediation model, when testing the effects of PYD on DASS dimensions, two of the PYD indicators (confidence and connection) emerged as significant predictors of life satisfaction, whereas life satisfaction significantly predicted all DASS dimensions. The PYD competence dimension was the only indicator which did not predict life satisfaction (see Table 4).

As expected, significant intercorrelations within PYD and DASS dimensions were observed in the partial mediation obtained model (see Figure 3), in which life satisfaction explained 33% of the total variance and the other endogenous variables explained 30% (anxiety), 33% (stress) and 47% (depression).

## 4. Discussion

The present study was developed with the main goal of analysing the relationship between competence, confidence and connection with stress, anxiety and depression, considering the mediator role of mental distress and life satisfaction. It was expected that the indicators of PYD would present a negative effect on mental distress and stress, anxiety and depression. It was also expected that the indicators of PYD would present a positive effect on life satisfaction. Finally, it was expected that mental distress and life satisfaction would mediate the relation between PYD indicators and stress, anxiety and depression. Two separate mediation analyses were carried out to understand the role of mental distress or life satisfaction on the relationship between PYD indicators and DASS dimensions, which confirmed the predicted relationships and partial mediations of mental distress and life satisfaction.

In fact, the results showed that adolescents with higher levels of competence, confidence and connection reported greater life satisfaction and lower mental distress. The obtained results also highlighted that adolescents with higher stress, depression and anxiety levels reported lower levels of competence, confidence and connection, as well as greater mental distress and lower life satisfaction. Higher levels of mental distress and lower life satisfaction were also shown to play a role in the relationship between competence, confidence and connection and stress, anxiety and depression, and were shown to contribute to these symptoms.

The obtained results confirmed that, in relation to the COVID-19 pandemic, psychological well-being decreased [1,2,3]. Also, a comparative meta-analysis has revealed that anxiety and depression increased among children and adolescents after COVID-19 [6]. Foster et al. [7] found that the stress experienced in the beginning of the COVID-19 pandemic had long-lasting impacts on mental health, including, specifically, an increased risk of developing anxious and depressive symptoms.

The results obtained in this study confirm the relevance and extent of this vulnerability. They not only confirm the findings of the previous literature, but also open a window of opportunities relating to transformable situations, such as promoting competence, confidence, and connection, which puts us in a possible trajectory of intervention.

The obtained results also confirm features already reported in the literature outlining the main risk factors, which include, among other factors, being female [5,6,9]. These indicators seem more pronounced in girls [8] and older students, who represent, in the literature, the groups presenting more psychological symptoms, lower life satisfaction, lower quality of life and fewer social–emotional skills [2,10,12]. Gender is therefore a concern to include in programmes aimed at promoting psychological health, given this identified vulnerability. On the other hand, the literature on positive youth development [15], which focuses on the ability to prevent mental health issues and promote psychological well-being, has found also that girls have higher levels of connectedness, while boys tend to have higher levels of competence and confidence and better psychological adjustment [16,18].

Despite the nature of the sample, this study has limitations related to its cross-sectional design, which allowed for the study of causality effects based on theoretical and intervention models. Also, the mediator models were studied using the total sample and decisions were taken based on the results from the literature with normative samples. Based on these limitations, we suggest that future studies assess longitudinal relationships between the studied variables, giving consideration to gender differences. The impact of early prevention and intervention evidence-based strategies, directed at the promotion of PYD, should also be studied.

Thus, these results show that it is crucial to identify and implement early prevention and intervention strategies, taking diversity—or, at least, gender diversity—into consideration. The results also indicate the importance of targeting to promote social emotional competence, such as connectedness, competence and confidence, to prevent mental unwellness, such as stress and anxious and depressive symptoms, either directly or by increasing perception of life satisfaction and lowering mental distress. In this context, results of one recent systematic literature review pointed to the importance of mindfulness-based interventions in schools for improving, among other factors, social–emotional competences and student well-being [33].

These results suggest that, in order to be effective, public policies regarding young people’s health and education would benefit from involving both targeted and indicated prevention strategies, including school and community-based interventions. Treatment would improve if a stepwise approach was followed, beginning with psychosocial interventions, followed, if needed, by specific therapy, and, finally, if needed, medication [34].

## 5. Conclusions

The promotion of connectedness, competence and confidence is related to better life satisfaction and a decrease in mental unwellness in adolescence. On the other hand, increased life satisfaction and decreased mental unwellness are associated with a lower frequency of depression, anxiety and stress. Finally, girls and older adolescents seem more prone to exhibiting mental unwellness and need more intense attention concerning their lifeworlds.

## Figures and Tables

**Table 1:** Descriptive statistics.

Variable	Level of Measurement	Range	M	SD	Skewness	Kurtosis	Cronbach’s Alpha
PYD							
Competence	Interval	0–24	13.55	4.67	−0.20	0.11	0.82
Confidence	Interval	0–24	14.37	5.60	−0.42	−0.32	0.90
Connection	Interval	0–32	20.13	5.79	−0.34	0.24	0.85
DASS							
Stress	Interval	0–21	5.69	4.76	0.86	0.17	0.89
Depression	Interval	0–21	5.03	4.87	1.13	0.66	0.90
Anxiety	Interval	0–21	4.21	4.39	1.30	1.19	0.86
Mental distress	Interval	0–25	14.39	5.32	−0.24	−0.50	0.82
Life satisfaction	Interval	0–10	7.05	1.85	−0.64	0.61	-

**Table 2:** Relationships between DASS dimensions with PYD dimensions, life satisfaction and mental distress.

	Stress	Depression	Anxiety	Life Satisfaction	Mental Distress
Competence	−0.41 ***	−0.49 ***	−0.42 ***	0.43 ***	0.51 ***
Confidence	−0.50 ***	−0.61 ***	−0.50 ***	0.51 ***	0.55 ***
Connection	−0.44 ***	−0.51 ***	−0.41 ***	0.52 ***	0.54 ***
Life satisfaction	−0.47 ***	−0.56 ***	−0.43 ***	-	-
Mental distress	−0.56 ***	−0.60 ***	−0.49 ***	0.58 ***	-

*** *p* ≤ 0.001.

**Table 3:** Direct, indirect and total effects for the mental distress mediator model.

					95% CI	
Direct Effects	Estimate	SE	Z	*p*	Lower	Upper
Competence -> Stress	0.001	0.005	−0.22	0.823	−0.010	0.008
Confidence -> Stress	−0.045	0.004	−11.06	<0.001	−0.053	−0.036
Connection -> Stress	−0.013	0.004	−3.62	<0.001	−0.020	−0.006
Competence -> Depression	−0.004	0.004	−0.97	0.332	−0.012	−0.004
Confidence -> Depression	−0.063	0.004	−16.88	<0.001	−0.070	−0.055
Connection -> Depression	−0.018	0.003	−5.68	<0.001	−0.025	−0.012
Competence -> Anxiety	−0.014	0.005	−2.89	0.004	−0.023	−0.004
Confidence -> Anxiety	−0.049	0.004	−11.61	<0.001	−0.058	−0.004
Connection -> Anxiety	−0.008	0.004	−2.16	0.030	−0.016	−0.000
Indirect Effects						
Competence -> HBSC5 -> Stress	−0.011	0.002	−5.94	<0.001	−0.014	−0.007
Confidence -> HBSC5 -> Stress	−0.020	0.002	−11.12	<0.001	−0.023	−0.016
Connection -> HBSC5 -> Stress	−0.019	0.002	−12.18	<0.001	−0.022	−0.016
Competence -> HBSC5 -> Depression	−0.009	0.002	−5.93	<0.001	−0.012	−0.006
Confidence -> HBSC5 -> Depression	−0.017	0.002	−11.03	<0.001	−0.021	−0.014
Connection -> HBSC5 -> Depression	−0.017	0.001	−12.06	<0.001	−0.020	−0.014
Competence -> HBSC5 -> Anxiety	−0.008	0.001	−5.70	<0.001	−0.010	−0.005
Confidence -> HBSC5 -> Anxiety	−0.014	0.001	−9.74	<0.001	−0.017	−0.012
Connection -> HBSC5 -> Anxiety	−0.014	0.001	−10.42	<0.001	−0.017	−0.012
Total effects						
Competence -> Stress	−0.012	0.005	−2.38	0.017	−0.021	−0.002
Confidence -> Stress	−0.065	0.004	−15.32	<0.001	−0.073	−0.056
Connection -> Stress	−0.032	0.004	−8.85	<0.001	−0.039	−0.025
Competence -> Depression	−0.013	0.004	−3.04	0.002	−0.022	−0.005
Confidence -> Depression	−0.080	0.004	−20.88	<0.001	−0.088	−0.073
Connection -> Depression	−0.035	0.003	−10.75	<0.001	−0.042	−0.028
Competence -> Anxiety	−0.022	0.005	−4.40	<0.001	−0.031	−0.012
Confidence -> Anxiety	−0.064	0.004	−14.95	<0.001	−0.072	−0.055
Connection -> Anxiety	−0.022	0.004	−6.05	<0.001	−0.029	−0.015

**Table 4:** Direct, indirect and total effects for the life satisfaction mediator model.

					95% CI	
Direct Effects	Estimate	SE	Z	*p*	Lower	Upper
Competence -> Stress	0.01	0.005	−2.02	0.044	−0.019	−0.000
Confidence -> Stress	−0.05	0.004	−12.37	<0.001	−0.060	0.044
Connection -> Stress	−0.02	0.004	−4.91	<0.001	−0.025	−0.011
Competence -> Depression	−0.01	0.004	−2.60	0.009	−0.019	−0.003
Confidence -> Depression	−0.07	0.004	−17.34	<0.001	−0.072	−0.057
Connection -> Depression	−0.02	0.003	−5.61	<0.001	−0.025	−0.012
Competence -> Anxiety	−0.02	0.005	−4.13	<0.001	−0.029	−0.010
Confidence -> Anxiety	−0.05	0.004	−12.44	<0.001	−0.062	−0.045
Connection -> Anxiety	−0.01	0.004	−2.86	0.004	−0.018	−0.003
Indirect Effects						
Competence -> HBSC5 -> Stress	−0.002	0.001	−1.78	0.076	−0.004	−0.000
Confidence -> HBSC5 -> Stress	−0.013	0.001	−9.17	<0.001	−0.015	−0.010
Connection -> HBSC5 -> Stress	−0.014	0.001	−10.28	<0.001	−0.017	−0.011
Competence -> HBSC5 -> Depression	−0.003	0.001	−1.78	0.074	−0.005	−0.000
Confidence -> HBSC5 -> Depression	−0.016	0.001	−10.93	<0.001	−0.018	−0.013
Connection -> HBSC5 -> Depression	−0.017	0.001	−12.12	<0.001	−0.020	−0.014
Competence -> HBSC5 -> Anxiety	−0.002	0.000	−1.77	0.077	−0.004	−0.000
Confidence -> HBSC5 -> Anxiety	−0.010	0.001	−8.16	<0.001	−0.013	−0.008
Connection -> HBSC5 -> Anxiety	−0.011	0.001	−8.92	<0.001	−0.014	−0.009
Total effects						
Competence -> Stress	−0.012	0.005	−2.39	0.017	−0.021	−0.002
Confidence -> Stress	−0.065	0.004	−15.33	<0.001	−0.073	−0.056
Connection -> Stress	−0.032	0.004	−8.82	<0.001	−0.039	−0.025
Competence -> Depression	−0.013	0.004	−3.04	0.002	−0.022	−0.005
Confidence -> Depression	−0.080	0.004	−20.90	<0.001	−0.088	−0.073
Connection -> Depression	−0.035	0.003	−10.72	<0.001	−0.042	−0.029
Competence -> Anxiety	−0.022	0.005	−4.41	<0.001	−0.031	−0.012
Confidence -> Anxiety	−0.064	0.004	−14.96	<0.001	−0.072	−0.055
Connection -> Anxiety	−0.022	0.004	−6.03	<0.001	−0.029	−0.015

## Data Availability

All data and materials are available upon request from Nuno Neto Rodrigues, data bank manager, due to privacy and ethical reasons.
